# Supplementary material for: Divergent east-west lineages in an Australian fruit fly, (Bactrocera jarvisi), associated with the Carpentaria Basin divide
Source: PLoS One. 2023 Jun 2;18(6):e0276247. doi: 10.1371/journal.pone.0276247 (PMC10237467; doi:10.1371/journal.pone.0276247)
Supplement: S1 Fig — (Kinship coefficient: 0.5 = monozygotic twins, 0.25 = full siblings, 0.125 = half siblings, 0.0 = unrelated). (DOCX) [file pone.0276247.s001.docx]

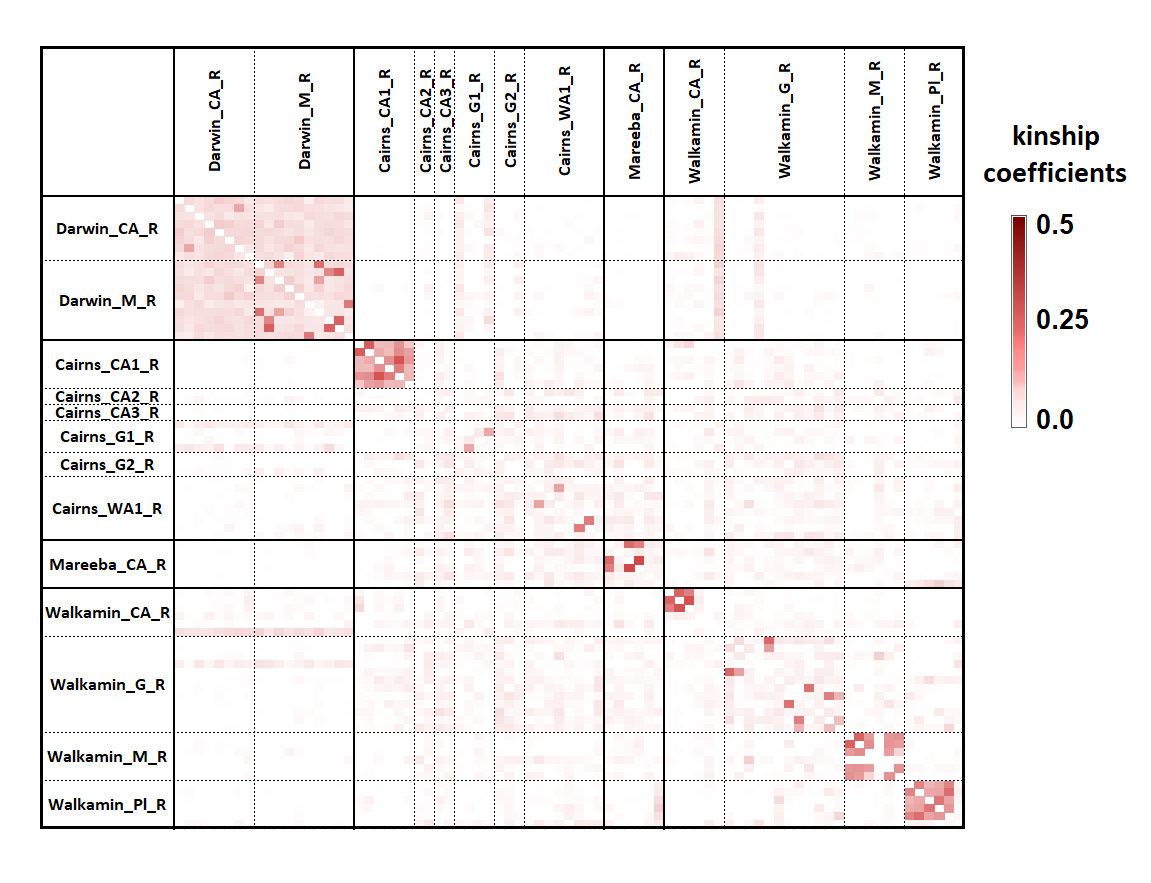


**S1 Fig. Heat map showing the pairwise kinship coefficients between the reared individuals. (Kinship coefficient: 0.5 = monozygotic twins, 0.25=full siblings, 0.125=half siblings, 0.0=unrelated)**
