# Supplementary material for: Divergent east-west lineages in an Australian fruit fly, (Bactrocera jarvisi), associated with the Carpentaria Basin divide
Source: PLoS One. 2023 Jun 2;18(6):e0276247. doi: 10.1371/journal.pone.0276247 (PMC10237467; doi:10.1371/journal.pone.0276247)
Supplement: S2 Fig — (DOCX) [file pone.0276247.s002.docx]

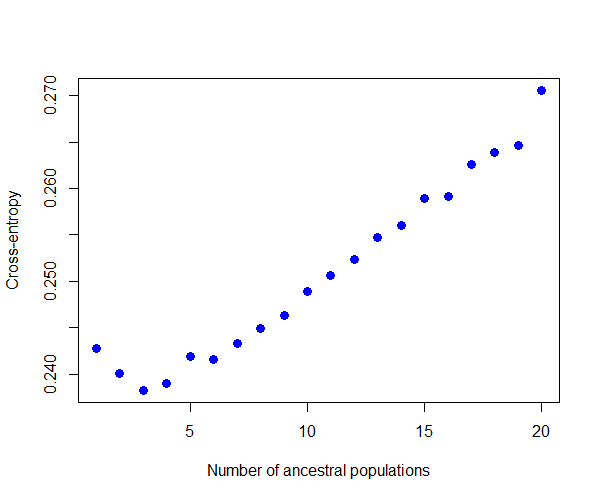


**S2 Fig. Plot of the cross-entropy values obtained from the sNMF algorithm in package LEA for K = 1–20, with 100 repetitions per each K value**
