# Supplementary material for: Divergent east-west lineages in an Australian fruit fly, (Bactrocera jarvisi), associated with the Carpentaria Basin divide
Source: PLoS One. 2023 Jun 2;18(6):e0276247. doi: 10.1371/journal.pone.0276247 (PMC10237467; doi:10.1371/journal.pone.0276247)
Supplement: S3 Fig — Each colour of the tip labels (sample IDs) corresponds with the location colours given in Fig 1. The numbers at each node represent the ultrafast-bootstrap support. (DOCX) [file pone.0276247.s003.docx]

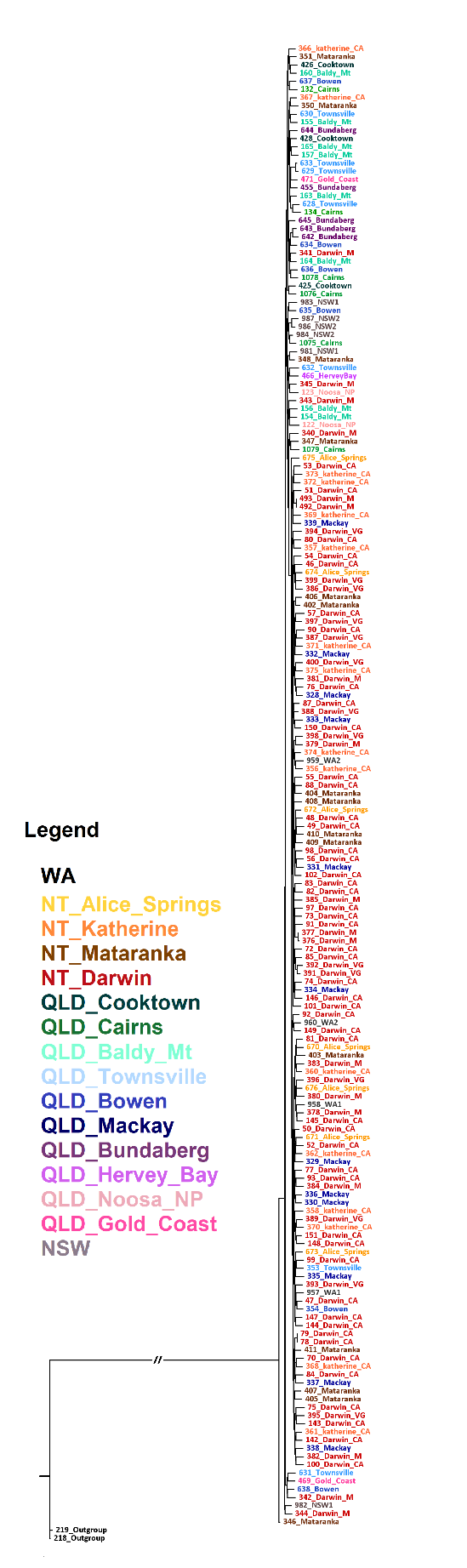


**S3 Fig. SNP maximum likelihood (IQ-TREE) consensus tree from 10,000 ultrafast-bootstrap replicates showing the true branch lengths. Each colour of the tip labels (sample IDs) corresponds with the location colours given in Fig. 1. The numbers at each node represent the ultrafast-bootstrap support.**
