# Supplementary material for: Divergent east-west lineages in an Australian fruit fly, (Bactrocera jarvisi), associated with the Carpentaria Basin divide
Source: PLoS One. 2023 Jun 2;18(6):e0276247. doi: 10.1371/journal.pone.0276247 (PMC10237467; doi:10.1371/journal.pone.0276247)
Supplement: S1 Table — (Sampling method = T: Trapping, R: Rearing). (DOCX) [file pone.0276247.s004.docx]

**S1 Table. Details of the samples used in the study. (Sampling method= T: Trapping, R: Rearing)**

| **State** | **Location** | **Population** | **Latitude** | **Longitude** | **Sampling method** | **Trap location/Host** | **Sample_ID** |
| --- | --- | --- | --- | --- | --- | --- | --- |
| **WA** | Kununurra | Kununurra_1 | -16.30908 | 128.24797 | T | T | 957 |
|  |  | Kununurra_1 | -16.30908 | 128.24797 | T | T | 958 |
|  |  | Kununurra_2 | -16.30861 | 128.24544 | T | T | 959 |
|  |  | Kununurra_2 | -16.30861 | 128.24544 | T | T | 960 |
| **NT** | **Alice Springs** | Alice Springs | -23.69837 | 133.87406 | T | Unknown | 670 |
|  |  | Alice Springs | -23.69837 | 133.87406 | T | Unknown | 671 |
|  |  | Alice Springs | -23.69837 | 133.87406 | T | Unknown | 672 |
|  |  | Alice Springs | -23.69837 | 133.87406 | T | Unknown | 673 |
|  |  | Alice Springs | -23.69837 | 133.87406 | T | Unknown | 674 |
|  |  | Alice Springs | -23.69837 | 133.87406 | T | Unknown | 675 |
|  |  | Alice Springs | -23.69837 | 133.87406 | T | Unknown | 676 |
|  | **Katherine** | Katherine | -14.46331 | 132.26017 | T | Cocky apple tree | 356 |
|  |  | Katherine | -14.46331 | 132.26017 | T | Cocky apple tree | 357 |
|  |  | Katherine | -14.46331 | 132.26017 | T | Cocky apple tree | 358 |
|  |  | Katherine | -14.46331 | 132.26017 | T | Cocky apple tree | 366 |
|  |  | Katherine | -14.46331 | 132.26017 | T | Cocky apple tree | 368 |
|  |  | Katherine | -14.46331 | 132.26017 | T | Cocky apple tree | 369 |
|  |  | Katherine | -14.46331 | 132.26017 | T | Cocky apple tree | 370 |
|  |  | Katherine | -14.46331 | 132.26017 | T | Cocky apple tree | 360 |
|  |  | Katherine | -14.46331 | 132.26017 | T | Cocky apple tree | 361 |
|  |  | Katherine | -14.46331 | 132.26017 | T | Cocky apple tree | 362 |
|  |  | Katherine | -14.46331 | 132.26017 | T | Cocky apple tree | 367 |
|  |  | Katherine | -14.46331 | 132.26017 | T | Cocky apple tree | 371 |
|  |  | Katherine | -14.46331 | 132.26017 | T | Cocky apple tree | 372 |
|  |  | Katherine | -14.46331 | 132.26017 | T | Cocky apple tree | 373 |
|  |  | Katherine | -14.46331 | 132.26017 | T | Cocky apple tree | 374 |
|  |  | Katherine | -14.46331 | 132.26017 | T | Cocky apple tree | 375 |
|  | **Mataranka** | Mataranka | -14.91901 | 133.06805 | T | Unknown | 402 |
|  |  | Mataranka | -14.91901 | 133.06805 | T | Unknown | 403 |
|  |  | Mataranka | -14.91901 | 133.06805 | T | Unknown | 404 |
|  |  | Mataranka | -14.91901 | 133.06805 | T | Unknown | 405 |
|  |  | Mataranka | -14.91901 | 133.06805 | T | Unknown | 406 |
|  |  | Mataranka | -14.91901 | 133.06805 | T | Unknown | 407 |
|  |  | Mataranka | -14.91901 | 133.06805 | T | Unknown | 408 |
|  |  | Mataranka | -14.91901 | 133.06805 | T | Unknown | 409 |
|  |  | Mataranka | -14.91901 | 133.06805 | T | Unknown | 410 |
|  |  | Mataranka | -14.91901 | 133.06805 | T | Unknown | 411 |
|  |  | Mataranka | -14.91901 | 133.06805 | T | Unknown | 346 |
|  |  | Mataranka | -14.91901 | 133.06805 | T | Unknown | 347 |
|  |  | Mataranka | -14.91901 | 133.06805 | T | Unknown | 348 |
|  |  | Mataranka | -14.91901 | 133.06805 | T | Unknown | 350 |
|  |  | Mataranka | -14.91901 | 133.06805 | T | Unknown | 351 |
|  | **Darwin** | Darwin_CA1 | -12.45438 | 130.94682 | T | Cocky apple tree | 100 |
|  |  | Darwin_CA1 | -12.45438 | 130.94682 | T | Cocky apple tree | 101 |
|  |  | Darwin_CA1 | -12.45438 | 130.94682 | T | Cocky apple tree | 102 |
|  |  | Darwin_CA1 | -12.45438 | 130.94682 | T | Cocky apple tree | 46 |
|  |  | Darwin_CA1 | -12.45438 | 130.94682 | T | Cocky apple tree | 47 |
|  |  | Darwin_CA1 | -12.45438 | 130.94682 | T | Cocky apple tree | 48 |
|  |  | Darwin_CA1 | -12.45438 | 130.94682 | T | Cocky apple tree | 49 |
|  |  | Darwin_CA1 | -12.45438 | 130.94682 | T | Cocky apple tree | 97 |
|  |  | Darwin_CA1 | -12.45438 | 130.94682 | T | Cocky apple tree | 98 |
|  |  | Darwin_CA1 | -12.45438 | 130.94682 | T | Cocky apple tree | 99 |
|  |  | Darwin_CA2 | -12.45438 | 130.94705 | T | Cocky apple tree | 51 |
|  |  | Darwin_CA2 | -12.45438 | 130.94705 | T | Cocky apple tree | 52 |
|  |  | Darwin_CA2 | -12.45438 | 130.94705 | T | Cocky apple tree | 53 |
|  |  | Darwin_CA2 | -12.45438 | 130.94705 | T | Cocky apple tree | 50 |
|  |  | Darwin_CA3 | -12.49703 | 130.96808 | T | Cocky apple tree | 54 |
|  |  | Darwin_CA3 | -12.49703 | 130.96808 | T | Cocky apple tree | 55 |
|  |  | Darwin_CA3 | -12.49703 | 130.96808 | T | Cocky apple tree | 56 |
|  |  | Darwin_CA3 | -12.49703 | 130.96808 | T | Cocky apple tree | 57 |
|  |  | Darwin_CA5 | -12.46181 | 130.98495 | T | Cocky apple tree | 75 |
|  |  | Darwin_CA5 | -12.46181 | 130.98495 | T | Cocky apple tree | 76 |
|  |  | Darwin_CA5 | -12.46181 | 130.98495 | T | Cocky apple tree | 77 |
|  |  | Darwin_CA5 | -12.46181 | 130.98495 | T | Cocky apple tree | 78 |
|  |  | Darwin_CA5 | -12.46181 | 130.98495 | T | Cocky apple tree | 79 |
|  |  | Darwin_CA6 | -12.49696 | 131.01326 | T | Cocky apple tree | 80 |
|  |  | Darwin_CA6 | -12.49696 | 131.01326 | T | Cocky apple tree | 81 |
|  |  | Darwin_CA6 | -12.49696 | 131.01326 | T | Cocky apple tree | 82 |
|  |  | Darwin_CA6 | -12.49696 | 131.01326 | T | Cocky apple tree | 83 |
|  |  | Darwin_CA6 | -12.49696 | 131.01326 | T | Cocky apple tree | 84 |
|  |  | Darwin_CA7 | -12.47578 | 130.94682 | T | Cocky apple tree | 85 |
|  |  | Darwin_CA7 | -12.47578 | 131.03443 | T | Cocky apple tree | 87 |
|  |  | Darwin_CA7 | -12.47578 | 131.03443 | T | Cocky apple tree | 88 |
|  |  | Darwin_CA8 | -12.57767 | 131.12604 | T | Cocky apple tree | 142 |
|  |  | Darwin_CA8 | -12.57767 | 131.12604 | T | Cocky apple tree | 143 |
|  |  | Darwin_CA8 | -12.57767 | 131.12604 | T | Cocky apple tree | 144 |
|  |  | Darwin_CA8 | -12.57767 | 131.12604 | T | Cocky apple tree | 145 |
|  |  | Darwin_CA8 | -12.57767 | 131.12604 | T | Cocky apple tree | 146 |
|  |  | Darwin_CA8 | -12.57767 | 131.12604 | T | Cocky apple tree | 147 |
|  |  | Darwin_CA8 | -12.57767 | 131.12604 | T | Cocky apple tree | 148 |
|  |  | Darwin_CA8 | -12.57767 | 131.12604 | T | Cocky apple tree | 149 |
|  |  | Darwin_CA8 | -12.57767 | 131.12604 | T | Cocky apple tree | 150 |
|  |  | Darwin_CA8 | -12.57767 | 131.12604 | T | Cocky apple tree | 151 |
|  |  | Darwin_CA9 | -12.60003 | 131.22304 | T | Cocky apple tree | 70 |
|  |  | Darwin_CA9 | -12.60003 | 131.22304 | T | Cocky apple tree | 71 |
|  |  | Darwin_CA9 | -12.60003 | 131.22304 | T | Cocky apple tree | 72 |
|  |  | Darwin_CA9 | -12.60003 | 131.22304 | T | Cocky apple tree | 73 |
|  |  | Darwin_CA9 | -12.60003 | 131.22304 | T | Cocky apple tree | 74 |
|  |  | Darwin_CA10 | -12.60814 | 131.26507 | T | Cocky apple tree | 90 |
|  |  | Darwin_CA10 | -12.60814 | 131.26507 | T | Cocky apple tree | 91 |
|  |  | Darwin_CA10 | -12.60814 | 131.26507 | T | Cocky apple tree | 92 |
|  |  | Darwin_CA10 | -12.60814 | 131.26507 | T | Cocky apple tree | 93 |
|  |  | Darwin_M1 | -12.58979 | 131.24660 | T | Mango Orchard | 492 |
|  |  | Darwin_M1 | -12.58979 | 131.24660 | T | Mango Orchard | 493 |
|  |  | Darwin_M1 | -12.58979 | 131.24660 | T | Mango Orchard | 340 |
|  |  | Darwin_M1 | -12.58979 | 131.24660 | T | Mango Orchard | 341 |
|  |  | Darwin_M1 | -12.58979 | 131.24660 | T | Mango Orchard | 342 |
|  |  | Darwin_M1 | -12.58979 | 131.24660 | T | Mango Orchard | 343 |
|  |  | Darwin_M1 | -12.58979 | 131.24660 | T | Mango Orchard | 344 |
|  |  | Darwin_M1 | -12.58979 | 131.24660 | T | Mango Orchard | 345 |
|  |  | Darwin_M2 | -12.76074 | 131.10273 | T | Mango Orchard | 376 |
|  |  | Darwin_M2 | -12.76074 | 131.10273 | T | Mango Orchard | 377 |
|  |  | Darwin_M2 | -12.76074 | 131.10273 | T | Mango Orchard | 378 |
|  |  | Darwin_M2 | -12.76074 | 131.10273 | T | Mango Orchard | 379 |
|  |  | Darwin_M2 | -12.76074 | 131.10273 | T | Mango Orchard | 380 |
|  |  | Darwin_M3 | -12.67877 | 131.06449 | T | Mango Orchard | 381 |
|  |  | Darwin_M3 | -12.67877 | 131.06449 | T | Mango Orchard | 382 |
|  |  | Darwin_M3 | -12.67877 | 131.06449 | T | Mango Orchard | 383 |
|  |  | Darwin_M3 | -12.67877 | 131.06449 | T | Mango Orchard | 384 |
|  |  | Darwin_M3 | -12.67877 | 131.06449 | T | Mango Orchard | 385 |
|  |  | Darwin_VG1 | -12.44500 | 130.92694 | T | VG_T | 386 |
|  |  | Darwin_VG1 | -12.44500 | 130.92694 | T | VG_T | 387 |
|  |  | Darwin_VG1 | -12.44500 | 130.92694 | T | VG_T | 388 |
|  |  | Darwin_VG1 | -12.44500 | 130.92694 | T | VG_T | 389 |
|  |  | Darwin_VG2 | -12.50444 | 130.98111 | T | VG_T | 391 |
|  |  | Darwin_VG2 | -12.50444 | 130.98111 | T | VG_T | 392 |
|  |  | Darwin_VG2 | -12.50444 | 130.98111 | T | VG_T | 393 |
|  |  | Darwin_VG2 | -12.50444 | 130.98111 | T | VG_T | 394 |
|  |  | Darwin_VG2 | -12.50444 | 130.98111 | T | VG_T | 395 |
|  |  | Darwin_VG3 | -12.59666 | 131.30416 | T | VG_T | 396 |
|  |  | Darwin_VG3 | -12.59666 | 131.30416 | T | VG_T | 397 |
|  |  | Darwin_VG3 | -12.59666 | 131.30416 | T | VG_T | 398 |
|  |  | Darwin_VG3 | -12.59666 | 131.30416 | T | VG_T | 399 |
|  |  | Darwin_VG3 | -12.59666 | 131.30416 | T | VG_T | 400 |
|  |  | Darwin_CA_R | N/A | N/A | R | Cocky apple fruit | 961 |
|  |  | Darwin_CA_R | N/A | N/A | R | Cocky apple fruit | 962 |
|  |  | Darwin_CA_R | N/A | N/A | R | Cocky apple fruit | 963 |
|  |  | Darwin_CA_R | N/A | N/A | R | Cocky apple fruit | 964 |
|  |  | Darwin_CA_R | N/A | N/A | R | Cocky apple fruit | 965 |
|  |  | Darwin_CA_R | N/A | N/A | R | Cocky apple fruit | 966 |
|  |  | Darwin_CA_R | N/A | N/A | R | Cocky apple fruit | 967 |
|  |  | Darwin_CA_R | N/A | N/A | R | Cocky apple fruit | 968 |
|  |  | Darwin_CA_R | N/A | N/A | R | Cocky apple fruit | 969 |
|  |  | Darwin_CA_R | N/A | N/A | R | Cocky apple fruit | 970 |
|  |  | Darwin_M_R | N/A | N/A | R | Mango fruit | 971 |
|  |  | Darwin_M_R | N/A | N/A | R | Mango fruit | 972 |
|  |  | Darwin_M_R | N/A | N/A | R | Mango fruit | 973 |
|  |  | Darwin_M_R | N/A | N/A | R | Mango fruit | 974 |
|  |  | Darwin_M_R | N/A | N/A | R | Mango fruit | 975 |
|  |  | Darwin_M_R | N/A | N/A | R | Mango fruit | 976 |
|  |  | Darwin_M_R | N/A | N/A | R | Mango fruit | 977 |
|  |  | Darwin_M_R | N/A | N/A | R | Mango fruit | 978 |
|  |  | Darwin_M_R | N/A | N/A | R | Mango fruit | 979 |
|  |  | Darwin_M_R | N/A | N/A | R | Mango fruit | 980 |
| **QLD** | **Cooktown** | Cooktown | -15.47581 | 145.24709 | T | T | 425 |
|  |  | Cooktown | -15.47581 | 145.24709 | T | T | 426 |
|  |  | Cooktown | -15.47581 | 145.24709 | T | T | 428 |
|  | **Cairns** | Cairns | -16.86030 | 145.77095 | T | T | 132 |
|  |  | Cairns | -16.86030 | 145.77095 | T | T | 133 |
|  |  | Cairns | -16.86030 | 145.77095 | T | T | 134 |
|  |  | Cairns_CA1_R | N/A | N/A | R | Cocky apple fruit | 930 |
|  |  | Cairns_CA1_R | N/A | N/A | R | Cocky apple fruit | 931 |
|  |  | Cairns_CA1_R | N/A | N/A | R | Cocky apple fruit | 932 |
|  |  | Cairns_CA1_R | N/A | N/A | R | Cocky apple fruit | 933 |
|  |  | Cairns_CA1_R | N/A | N/A | R | Cocky apple fruit | 934 |
|  |  | Cairns_CA1_R | N/A | N/A | R | Cocky apple fruit | 935 |
|  |  | Cairns_CA2_R | N/A | N/A | R | Cocky apple fruit | 949 |
|  |  | Cairns_CA2_R | N/A | N/A | R | Cocky apple fruit | 950 |
|  |  | Cairns_CA3_R | N/A | N/A | R | Cocky apple fruit | 951 |
|  |  | Cairns_CA3_R | N/A | N/A | R | Cocky apple fruit | 952 |
|  |  | Cairns_G1_R | N/A | N/A | R | Guava fruit | 677 |
|  |  | Cairns_G1_R | N/A | N/A | R | Guava fruit | 678 |
|  |  | Cairns_G1_R | N/A | N/A | R | Guava fruit | 679 |
|  |  | Cairns_G1_R | N/A | N/A | R | Guava fruit | 955 |
|  |  | Cairns_G2_R | N/A | N/A | R | Guava fruit | 680 |
|  |  | Cairns_G2_R | N/A | N/A | R | Guava fruit | 681 |
|  |  | Cairns_G2_R | N/A | N/A | R | Guava fruit | 956 |
|  |  | Cairns_WA1_R | N/A | N/A | R | White bush apple fruit | 941 |
|  |  | Cairns_WA1_R | N/A | N/A | R | White bush apple fruit | 942 |
|  |  | Cairns_WA1_R | N/A | N/A | R | White bush apple fruit | 943 |
|  |  | Cairns_WA1_R | N/A | N/A | R | White bush apple fruit | 944 |
|  |  | Cairns_WA1_R | N/A | N/A | R | White bush apple fruit | 945 |
|  |  | Cairns_WA1_R | N/A | N/A | R | White bush apple fruit | 946 |
|  |  | Cairns_WA1_R | N/A | N/A | R | White bush apple fruit | 947 |
|  |  | Cairns_WA1_R | N/A | N/A | R | White bush apple fruit | 948 |
|  |  | Cairns3_P | N/A | N/A | T | Papaya tree | 1078 |
|  |  | Cairns3_P | N/A | N/A | T | Papaya tree | 1079 |
|  | **Mareeba** | Mareeba_CA | N/A | N/A | R | Cocky apple fruit | 924 |
|  |  | Mareeba_CA | N/A | N/A | R | Cocky apple fruit | 925 |
|  |  | Mareeba_CA | N/A | N/A | R | Cocky apple fruit | 926 |
|  |  | Mareeba_CA | N/A | N/A | R | Cocky apple fruit | 927 |
|  |  | Mareeba_CA | N/A | N/A | R | Cocky apple fruit | 928 |
|  |  | Mareeba_CA | N/A | N/A | R | Cocky apple fruit | 929 |
|  |  | Mareeba_M | N/A | N/A | T | Mango Orchard | 1075 |
|  |  | Mareeba_M | N/A | N/A | T | Mango Orchard | 1076 |
|  | **Walkamin** | Walkamin_CA | N/A | N/A | R | Cocky apple fruit | 937 |
|  |  | Walkamin_CA | N/A | N/A | R | Cocky apple fruit | 938 |
|  |  | Walkamin_CA | N/A | N/A | R | Cocky apple fruit | 939 |
|  |  | Walkamin_CA | N/A | N/A | R | Cocky apple fruit | 940 |
|  |  | Walkamin_CA_R | N/A | N/A | R | Cocky apple fruit | 953 |
|  |  | Walkamin_CA_R | N/A | N/A | R | Cocky apple fruit | 954 |
|  |  | Walkamin_G_R | N/A | N/A | R | Guava fruit | 900 |
|  |  | Walkamin_G_R | N/A | N/A | R | Guava fruit | 901 |
|  |  | Walkamin_G_R | N/A | N/A | R | Guava fruit | 902 |
|  |  | Walkamin_G_R | N/A | N/A | R | Guava fruit | 903 |
|  |  | Walkamin_G_R | N/A | N/A | R | Guava fruit | 904 |
|  |  | Walkamin_G_R | N/A | N/A | R | Guava fruit | 905 |
|  |  | Walkamin_G_R | N/A | N/A | R | Guava fruit | 906 |
|  |  | Walkamin_G_R | N/A | N/A | R | Guava fruit | 907 |
|  |  | Walkamin_G_R | N/A | N/A | R | Guava fruit | 908 |
|  |  | Walkamin_G_R | N/A | N/A | R | Guava fruit | 909 |
|  |  | Walkamin_G_R | N/A | N/A | R | Guava fruit | 910 |
|  |  | Walkamin_G_R | N/A | N/A | R | Guava fruit | 911 |
|  |  | Walkamin_M_R | N/A | N/A | R | Mango fruit | 912 |
|  |  | Walkamin_M_R | N/A | N/A | R | Mango fruit | 913 |
|  |  | Walkamin_M_R | N/A | N/A | R | Mango fruit | 914 |
|  |  | Walkamin_M_R | N/A | N/A | R | Mango fruit | 915 |
|  |  | Walkamin_M_R | N/A | N/A | R | Mango fruit | 916 |
|  |  | Walkamin_M_R | N/A | N/A | R | Mango fruit | 917 |
|  |  | Walkamin_Pl_R | N/A | N/A | R | Plum fruit | 918 |
|  |  | Walkamin_Pl_R | N/A | N/A | R | Plum fruit | 919 |
|  |  | Walkamin_Pl_R | N/A | N/A | R | Plum fruit | 920 |
|  |  | Walkamin_Pl_R | N/A | N/A | R | Plum fruit | 921 |
|  |  | Walkamin_Pl_R | N/A | N/A | R | Plum fruit | 922 |
|  |  | Walkamin_Pl_R | N/A | N/A | R | Plum fruit | 923 |
|  | **Baldy Mt** | Baldy Mt | -17.27281 | 145.46600 | T | T | 154 |
|  |  | Baldy Mt | -17.27281 | 145.46600 | T | T | 155 |
|  |  | Baldy Mt | -17.27281 | 145.46600 | T | T | 156 |
|  |  | Baldy Mt | -17.27281 | 145.46600 | T | T | 157 |
|  |  | Baldy Mt | -17.27281 | 145.46600 | T | T | 160 |
|  |  | Baldy Mt | -17.27281 | 145.46600 | T | T | 163 |
|  |  | Baldy Mt | -17.27281 | 145.46600 | T | T | 164 |
|  |  | Baldy Mt | -17.27281 | 145.46600 | T | T | 165 |
|  | **Townsville** | Townsville | -19.22269 | 146.67944 | T | T | 628 |
|  |  | Townsville | -19.22269 | 146.67944 | T | T | 629 |
|  |  | Townsville | -19.22269 | 146.67944 | T | T | 630 |
|  |  | Townsville | -19.22269 | 146.67944 | T | T | 631 |
|  |  | Townsville | -19.22269 | 146.67944 | T | T | 632 |
|  |  | Townsville | -19.22269 | 146.67944 | T | T | 633 |
|  |  | Townsville | -19.22269 | 146.67944 | T | T | 352 |
|  |  | Townsville | -19.22269 | 146.67944 | T | T | 353 |
|  | **Bowen** | Bowen | -20.01210 | 148.24627 | T | T | 634 |
|  |  | Bowen | -20.01210 | 148.24627 | T | T | 635 |
|  |  | Bowen | -20.01210 | 148.24627 | T | T | 636 |
|  |  | Bowen | -20.01210 | 148.24627 | T | T | 637 |
|  |  | Bowen | -20.01210 | 148.24627 | T | T | 638 |
|  |  | Bowen | -20.01210 | 148.24627 | T | T | 354 |
|  | **Mackay** | Mackay | -21.15106 | 149.19892 | T | T | 328 |
|  |  | Mackay | -21.15106 | 149.19892 | T | T | 329 |
|  |  | Mackay | -21.15106 | 149.19892 | T | T | 330 |
|  |  | Mackay | -21.15106 | 149.19892 | T | T | 331 |
|  |  | Mackay | -21.15106 | 149.19892 | T | T | 332 |
|  |  | Mackay | -21.15106 | 149.19892 | T | T | 333 |
|  |  | Mackay | -21.15106 | 149.19892 | T | T | 334 |
|  |  | Mackay | -21.15106 | 149.19892 | T | T | 335 |
|  |  | Mackay | -21.15106 | 149.19892 | T | T | 336 |
|  |  | Mackay | -21.15106 | 149.19892 | T | T | 337 |
|  |  | Mackay | -21.15106 | 149.19892 | T | T | 338 |
|  |  | Mackay | -21.15106 | 149.19892 | T | T | 339 |
|  | **Bundaberg** | Bundaberg | -24.85048 | 152.35064 | T | T | 455 |
|  |  | Bundaberg | -24.85048 | 152.35064 | T | T | 642 |
|  |  | Bundaberg | -24.85048 | 152.35064 | T | T | 643 |
|  |  | Bundaberg | -24.85048 | 152.35064 | T | T | 644 |
|  |  | Bundaberg | -24.85048 | 152.35064 | T | T | 645 |
|  | **Hervey Bay** | Hervey Bay | -25.30250 | 152.88861 | T | T | 466 |
|  | **Noosa NP** | Noosa NP | -26.38312 | 153.10032 | T | T | 122 |
|  |  | Noosa NP | -26.38312 | 153.10032 | T | T | 123 |
|  | **Gold Coast** | Gold Coast | -28.01666 | 153.43600 | T | T | 469 |
|  |  | Gold Coast | -28.01666 | 153.43600 | T | T | 471 |
| **NSW** | Tenterfield | Tenterfield | -29.05743 | 152.01846 | T | T | 981 |
|  |  | Tenterfield | -29.05743 | 152.01846 | T | T | 982 |
|  |  | Tenterfield | -29.05743 | 152.01846 | T | T | 983 |
|  | Alstonville | Alstonville | -28.84208 | 153.44035 | T | T | 984 |
|  |  | Alstonville | -28.84208 | 153.44035 | T | T | 985 |
|  |  | Alstonville | -28.84208 | 153.44035 | T | T | 986 |
|  |  | Alstonville | -28.84208 | 153.44035 | T | T | 987 |
|  |  | Alstonville | -28.84208 | 153.44035 | T | T | 988 |
|  |  | Alstonville | -28.84208 | 153.44035 | T | T | 989 |
|  |  | Alstonville | -28.84208 | 153.44035 | T | T | 990 |
